# Efficacy and Safety of Azvudine in Patients With COVID‐19 in China: A Meta‐Analysis of Observational Studies

**DOI:** 10.1111/crj.13798

**Published:** 2024-07-12

**Authors:** Tao Dong, Wentao Zhang, Tingting Wu, Yongxiang Ge, Qi Yang, Jia Xu, Yuna Liu

**Affiliations:** ^1^ Pharmacy Department Beijing Hospital of Integrated Traditional Chinese and Western Medicine Beijing China; ^2^ Medical Device Monitoring and Evaluation Department National Center for ADR Monitoring Beijing China; ^3^ Department of Infectious Diseases Beijing Hospital of Integrated Chinese and Western Medicine Beijing China; ^4^ Department of Clinical Laboratory Beijing Hospital of Integrated Chinese and Western Medicine Beijing China

**Keywords:** azvudine, COVID‐19, meta‐analysis, mortality, Paxlovid

## Abstract

**Background:**

Azvudine (FNC) is a novel small molecule antiviral drug for treating COVID‐19 that is available only on the Chinese market. Despite being recommended for treating COVID‐19 by the Chinese guidelines, its efficacy and safety are still unclear. This study aimed to evaluate the protective effect of FNC on COVID‐19 outcomes and its safety.

**Methods:**

We followed the PRISMA 2020 guidelines and searched the PubMed, Embase, Web of Science, Scopus, and China National Knowledge Infrastructure (CNKI) databases to evaluate studies on the effectiveness of FNC in treating COVID‐19 in China, focusing on mortality and overall outcomes. Additionally, its impact on the length of hospital stay (LOHS), time to first nucleic acid negative conversion (T‐FNANC), and adverse events was evaluated. The inclusion criterion was that the studies were published from July 2021 to April 10, 2024. This study uses the ROBINS‐I tool to assess bias risk and employs the GRADE approach to evaluate the certainty of the evidence.

**Results:**

The meta‐analysis included 24 retrospective studies involving a total of 11 830 patients. Low‐certainty evidence revealed no significant difference in mortality (OR = 0.91, 95% CI: 0.76–1.08) or LOHS (WMD = −0.24, 95% CI: −0.83 to 0.35) between FNC and Paxlovid in COVID‐19 patients. Low‐certainty evidence shows that the T‐FNANC was longer (WMD = 1.95, 95% CI: 0.36–3.53). Compared with the Paxlovid group, low‐certainty evidence shows the FNC group exhibited a worse composite outcome (OR = 0.77, 95% CI: 0.63–0.95) and fewer adverse events (OR = 0.63, 95% CI: 0.46–0.85). Compared with supportive treatment, low certainty shows FNC significantly reduced the mortality rate in COVID‐19 patients (OR = 0.61, 95% CI: 0.51–0.74) and decreased the composite outcome (OR = 0.67, 95% CI: 0.50–0.91), and very low certainty evidence shows significantly decreased the T‐FNANC (WMD = −4.62, 95% CI: −8.08 to −1.15). However, in very low certainty, there was no significant difference in LOHS (WMD = −0.70, 95% CI: −3.32 to 1.91) or adverse events (OR = 1.97, 95% CI: 0.48–8.17).

**Conclusions:**

FNC appears to be a safe and potentially effective treatment for COVID‐19 in China, but further research with larger, high‐quality studies is necessary to confirm these findings. Due to the certainty of the evidence and the specific context of the studies conducted in China, caution should be exercised when considering whether the results are applicable worldwide.

**Trial Registration:**

PROSPERO number: CRD42024520565

## Graphical abstract

We performed a meta‐analysis with 24 observational studies concerning the effect of Chinese COVID‐19 patients with azvudine. We discovered that azvudine offers a good protective effect and safety profile, significantly reducing mortality of COVID‐19 patients. However, the low certainty of the evidence leads us to adopt a cautious stance regarding this conclusion.

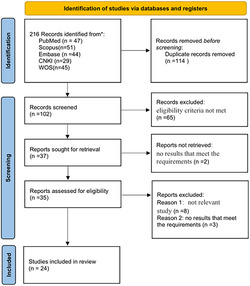

## 1 Introduction

COVID‐19, characterized by its high incidence and lack of specific antiviral drugs, has spread rapidly worldwide, posing a severe threat to human safety and health [1]. Antiviral therapy is an important treatment measure for COVID‐19, and initiating antiviral treatment as early as possible can effectively inhibit viral replication and control the progression of novel coronavirus infection, thus reducing mortality [2]. FNC is a broad‐spectrum RNA virus inhibitor that was initially used for the treatment of HIV infection [3]. Preclinical studies have shown that FNC can effectively inhibit the SARS‐CoV‐2 strain, improve lung damage, enhance immunity, and significantly shorten T‐FNANC [4, 5], and it also has a good safety profile. Premarketing clinical research has shown that FNC can shorten the T‐FNANC in patients with mild COVID‐19, and the incidence rate of adverse events is similar to that of a placebo [6]. The National Medical Products Administration of China conditionally approved FNC for the treatment of adult patients with mild COVID‐19 on July 25, 2022 [7]. Currently, there are still doubts about the safety and efficacy of FNC in treating COVID‐19. This study aimed to evaluate the protective effect of FNC on COVID‐19 patients in China by pooling clinical studies conducted in China.

## 2 Method

### 2.1 Retrieval Strategy and Literature Screening

We searched databases such as PubMed, Embase, Scopus, WOS and CNKI and retrieved literature published after July 2021. The latest search was conducted on April 10, 2024, using the search terms “azvudine,” “COVID‐19,” and “SARS‐CoV‐2.” As FNC is the latest drug approved for treating COVID‐19, we considered this search strategy to be sufficiently sensitive to capture all published literature on the impact of FNC on COVID‐19. Two investigators, Wt Zhang and T Dong, independently planned, implemented, and cross‐checked all the search strategies. There were no language restrictions for inclusion. To avoid overlooking any potentially relevant studies, investigators also reviewed the reference lists of selected papers. After filtering duplicate items using EndNote 9, the two researchers conducted back‐to‐back readings of the titles and abstracts of these articles for initial screening. They then examined the full text of any articles initially meeting the inclusion criteria, focusing on the hospitals, researchers, and subjects of the studies for careful analysis to avoid including duplicate studies, ensuring the accuracy and credibility of the analysis results. Any discrepancies were discussed with the corresponding author, Yn Liu, to reach a consensus.

Studies that met the following criteria were considered for inclusion: (1) were patients diagnosed with COVID‐19 infection; (2) used FNC as the experimental drug; (3) used Paxlovid or other antiviral drugs used as controls or symptomatic supportive treatment used as controls; (4) were observational, RCTs were excluded; and (5) were conducted in China. Preprints are considered eligible; if a preprint is subsequently published (before April 10, 2024), only the published version is included. All nonclinical studies, posters or conference abstracts, and studies lacking sufficient data for analysis of the results were excluded. We also excluded studies with ≤ 10 patients, studies conducted outside of China, studies for which the full text could not be obtained, and case reports.

### 2.2 Data Extraction and Results of Interest

The following study characteristics and demographic data were independently extracted by the two reviewers, Wt Zhang and T Dong: author, publication year, T‐FNANC, number of deaths, LOHS, composite outcome and adverse events. We extracted the means and standard deviations or medians and interquartile ranges for continuous variables.

The primary outcomes of interest were as follows: (a) mortality rate, defined as the COVID‐19‐specific or overall mortality rate within 28 or 30 days after a positive test result; (b) incidence of composite outcome, defined as the number of patients requiring invasive ventilation and/or admission to the ICU and/or death. Additionally, T‐FNANC, LOHS, and adverse events are of interest.

### 2.3 Quality Assessment and Risk of Bias

We assessed the risk of bias in the studies using the ROBINS‐I tool. Additionally, we evaluated publication bias using both Egger's test and a visual inspection of the symmetry in funnel plots. Publication bias was subjectively assessed through funnel plots and objectively through Egger's test at a 5% significance level. According to Egger's test, a *p* value less than 0.05 suggests the presence of publication bias, while a *p* value greater than 0.05 suggests its absence [8, 9]. However, for studies with fewer than nine items, we did not generate funnel plots to assess publication bias.

### 2.4 Quality of Evidence Evaluation

For each outcome, two authors (Wt Zhang and T Dong) independently assessed the quality of the evidence using the GRADE approach. The certainty of the evidences was graded as high, moderate, low, and very low.

### 2.5 Statistical Analysis

All analyses were conducted using Stata 17.0 software. For continuous variables, we estimated the mean and standard deviation (SD) values based on the median and range or quartiles provided in each study, as suggested by Wan et al. [10]. We analyzed continuous and dichotomous variables separately, considering the weighted mean difference (WMD) and odds ratio (OR) along with their 95% confidence intervals (CIs). A statistical significance threshold of 0.05 was set, and all reported CIs were 95% CIs. When *I* ^2^ > 50% and *p* < 0.05, significant heterogeneity was indicated, and a random‐effects model was used; when *I* ^2^ < 50% and *p* ≥ 0.05, indicating nonsignificant heterogeneity, a fixed‐effects model was used. Random‐effects and fixed‐effects models were applied to studies with high and low heterogeneity, respectively.

## 3 Results

### 3.1 Search Results

**FIGURE 1:**
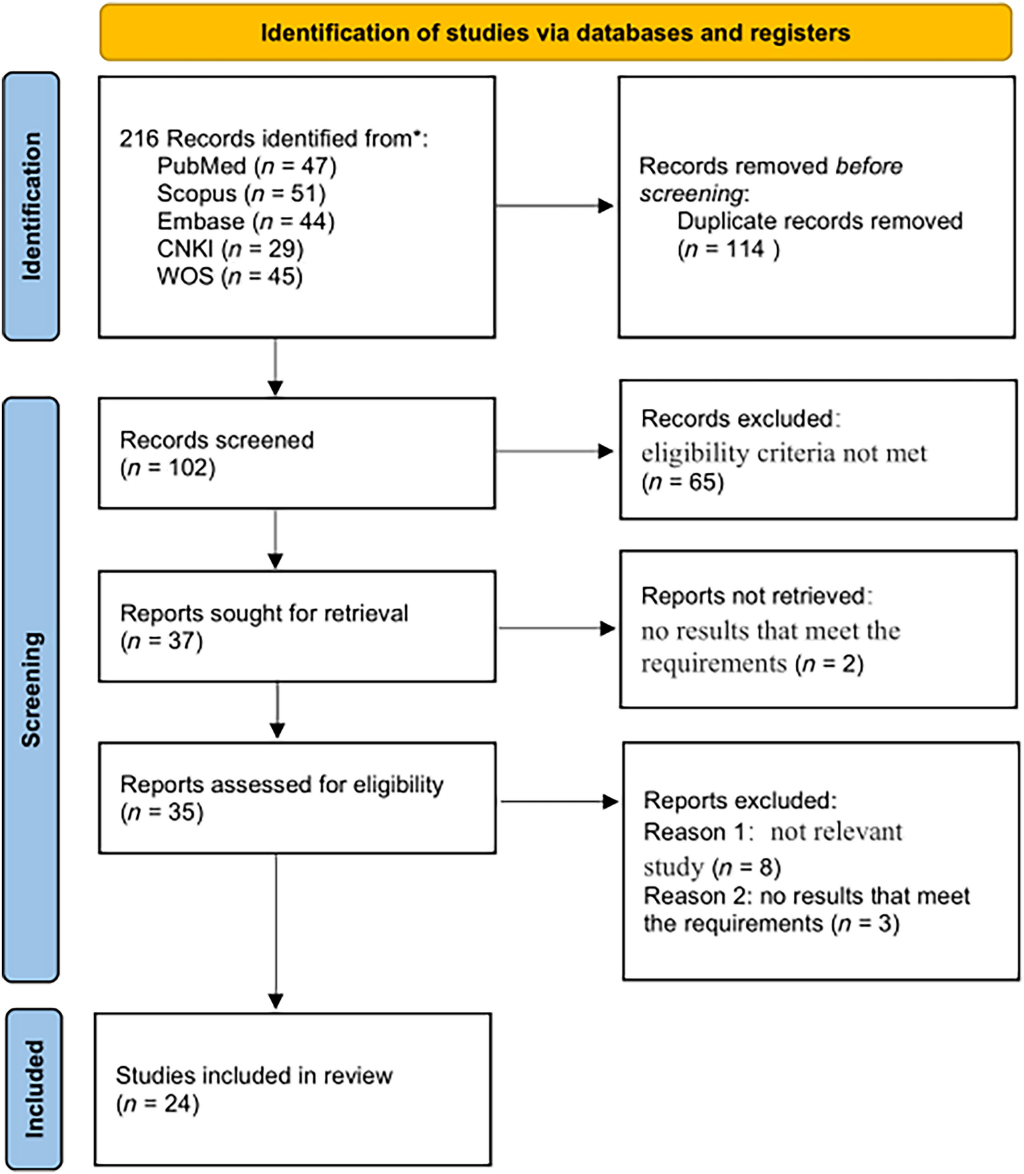
PRISMA flow diagram. *Consider, if feasible to do so, reporting the number of records identified from each database or register searched (rather than the total number across all databases/registers).

A total of 24 studies involving 11 830 patients was included in the meta‐analysis [11, 12, 13, 14, 15, 16, 17, 18, 19, 20, 21, 22, 23, 24, 25, 26, 27, 28, 29, 30, 31, 32, 33, 34]. The PRISMA flow chart is presented in Figure 1. Table 1 displays the basic characteristics of the included studies. Nine studies compared the efficacy and safety of FNC and Paxlovid in treating COVID‐19, 10 studies compared the efficacy and safety of FNC and supportive therapy, and 5 studies compared the efficacy and safety of FNC, Paxlovid, and supportive therapy in treating COVID‐19. These clinical studies were all conducted in China, with 22 published in English and 2 published in Chinese [28, 29]. In terms of disease severity, the majority of the included patients in most studies had mild to moderate disease, with four studies including patients with severe to critical illness [14, 23, 32, 33]. Among the analyzed population, one study focused on renal transplant recipients [28], one included individuals with a history of cancer [30], and one study included patients with concomitant cardiovascular disease [31].

**TABLE 1:** Characteristics of the included studies.

No.	Author	Publication year	Study population	No. of patients	Antiviral therapy	Outcomes
1	Zhou	2024	Older adults with mild or moderate COVID‐19	55	FNC/RN/SOC	T‐FNANC, composite outcome, AEs
2	Su	2024	Hospitalized patients with SARS‐CoV‐2 infection	171	FNC/RN	Mortality, LOHS, AEs
3	Han	2024	Hospitalized patients with SARS‐CoV‐2 infection	972	FNC/RN	Mortality, composite outcome, AEs
4	Zong	2023	Admitted for COVID‐19 pneumonia	585	FNC/SOC	Mortality
5	Wei	2023	Hospitalized adult patients (aged ≥ 18 years) with COVID‐19	725	FNC/RN	Mortality, composite outcome, AEs
6	Zhao X	2023	Patients with COVID‐19	227	FNC/RN	T‐FNANC, mortality, LHOS
7	Shang	2023	Hemodialysis patients infected with COVID‐19	364	FNC/SOC	AEs
8	Liu	2023	Patients hospitalized confirmed COVID‐19	1110	FNC/SOC	Mortality
9	Yang	2023	Nonhospitalized patients who tested positive for SARS‐CoV‐2	804	FNC/SOC	Mortality, AEs
10	Dian	2023	SARS‐CoV‐2 infection	456	FNC/RN	Mortality, composite outcome
11	Zhao Qq	2023	Hospitalized patients with COVID‐19	281	FNC/RN	T‐FNANC, LOHS, composite outcome, AEs
12	Shao	2023	Severely and critically ill patients with COVID‐19	966	FNC/RN/SOC	Mortality
13	Gao	2023	Hospitalized patients with COVID‐19	134	FNC/RN	T‐FNANC
14	Sun	2023	Hospitalized patients with COVID‐19	490	FNC/SOC	Mortality, composite outcome
15	Deng	2023	Hospitalized COVID‐19 patients	562	FNC/RN	Mortality, composite outcome
16	Chen	2023	Patients with a diagnosis of COVID‐19	207	FNC/SOC	T‐FNANC, LOHS
17	Hu	2023	Patients hospitalized owing to SARS‐CoV‐2 infection	535	FNC/RN/SOC	Mortality
18	Zhang	2023	Mild to moderate hospitalized patients infected with COVID‐19	340	FNC/SOC	LOHS, composite outcome
19	Yang	2023	Moderate COVID‐19 in kidney transplant recipient	65	FNC/SOC	T‐FNANC, composite outcome, AEs
20	Zhou	2023	Hospitalized adults with moderate‐to‐severe COVID‐19	1154	FNC/RN/SOC	T‐FNANC, mortality, composite outcome, AEs
21	Li	2024	Hospitalized patients with COVID‐19 and preexisting cancer	84	FNC/SOC	T‐FNANC, LOHS
22	Wu	2024	Patients with confirmed COVID‐19 and preexisting cardiovascular diseases	347	FNC/SOC	Mortality
23	Wang	2024	Elderly patients with severe COVID‐19 infection	241	FNC/RN/SOC	Mortality, composite outcome
24	Zhang	2024	Severe to critical COVID‐19 inpatients	955	FNC/RN	Mortality, LOHS

### 3.2 Characteristics of Included Studies

The methodological quality of the studies included in the meta‐analysis was deemed acceptable. We utilized the ROBINS‐I tool [35] to assess the quality of all studies. None of the observational studies were deemed to have a low risk of bias, with most studies categorized as having moderate bias, but only Shao's study was considered to have serious bias. Importantly, all 24 studies met the predefined inclusion criteria. These results are summarized in Table 2.

**TABLE 2:** Quality assessment of the studies.

Author	Year	Bias due to confounding	Bias in selection of participants into the study	Bias in classification of interventions	Bias due to deviations from intended interventions	Bias due to missing data	Bias in measurement of outcomes	Bias in selection of the reported result	Overall bias
Zhou	2024	Moderate	Low	Low	Low	Low	Low	Moderate	Moderate
Su	2024	Moderate	Low	Low	Low	Low	Low	Moderate	Moderate
Han	2024	Moderate	Low	Low	Low	Low	Low	Moderate	Moderate
Zong	2023	Moderate	Low	Low	Low	Low	Low	Moderate	Moderate
Wei	2023	Moderate	Low	Low	Low	Low	Low	Moderate	Moderate
Zhao X	2023	Moderate	Low	Low	Low	Low	Low	Moderate	Moderate
Liu	2023	Moderate	Low	Low	Low	Low	Low	Moderate	Moderate
Yang	2023	Moderate	Low	Low	Low	Low	Low	Moderate	Moderate
Dian	2023	Moderate	Low	Low	Low	Low	Low	Moderate	Moderate
Zhao Qq	2023	Moderate	Low	Low	Low	Low	Low	Moderate	Moderate
Shao	2023	Serious	Low	Low	Low	Low	Low	Moderate	Serious
Gao	2023	Moderate	Low	Low	Low	Low	Low	Moderate	Moderate
Sun	2023	Moderate	Low	Low	Low	Low	Low	Moderate	Moderate
Deng	2023	Moderate	Low	Low	Low	Low	Low	Moderate	Moderate
Hu	2023	Moderate	Low	Low	Low	Low	Low	Moderate	Moderate
Zhang	2023	Moderate	Low	Low	Low	Low	Low	Moderate	Moderate
Yang	2023	Moderate	Low	Low	Low	Low	Low	Moderate	Moderate
Zhou	2023	Moderate	Low	Low	Low	Low	Low	Moderate	Moderate
Li	2024	Moderate	Low	Low	Low	Low	Moderate	Moderate	Moderate
Liu	2024	Moderate	Low	Low	Low	Low	Moderate	Moderate	Moderate
Wang	2024	Moderate	Low	Low	Low	Low	Moderate	Moderate	Moderate
Zhou	2023	Moderate	Low	Low	Low	Low	Low	Low	Moderate
Shang	2023	Moderate	Low	Low	Moderate	Low	Low	Low	Moderate
Zhang	2024	Moderate	Low	Low	Low	Moderate	Low	Low	Moderate

Appendix S1 provides a table summarizing the conclusions and the certainty of the evidence based on the GRADE approach [36]. Since all the included studies were retrospective, and some of the study results had excessively wide confidence intervals, the certainty of most of the results is low, and the certainty of a few results is very low.

### 3.3 Mortality

**FIGURE 2:**
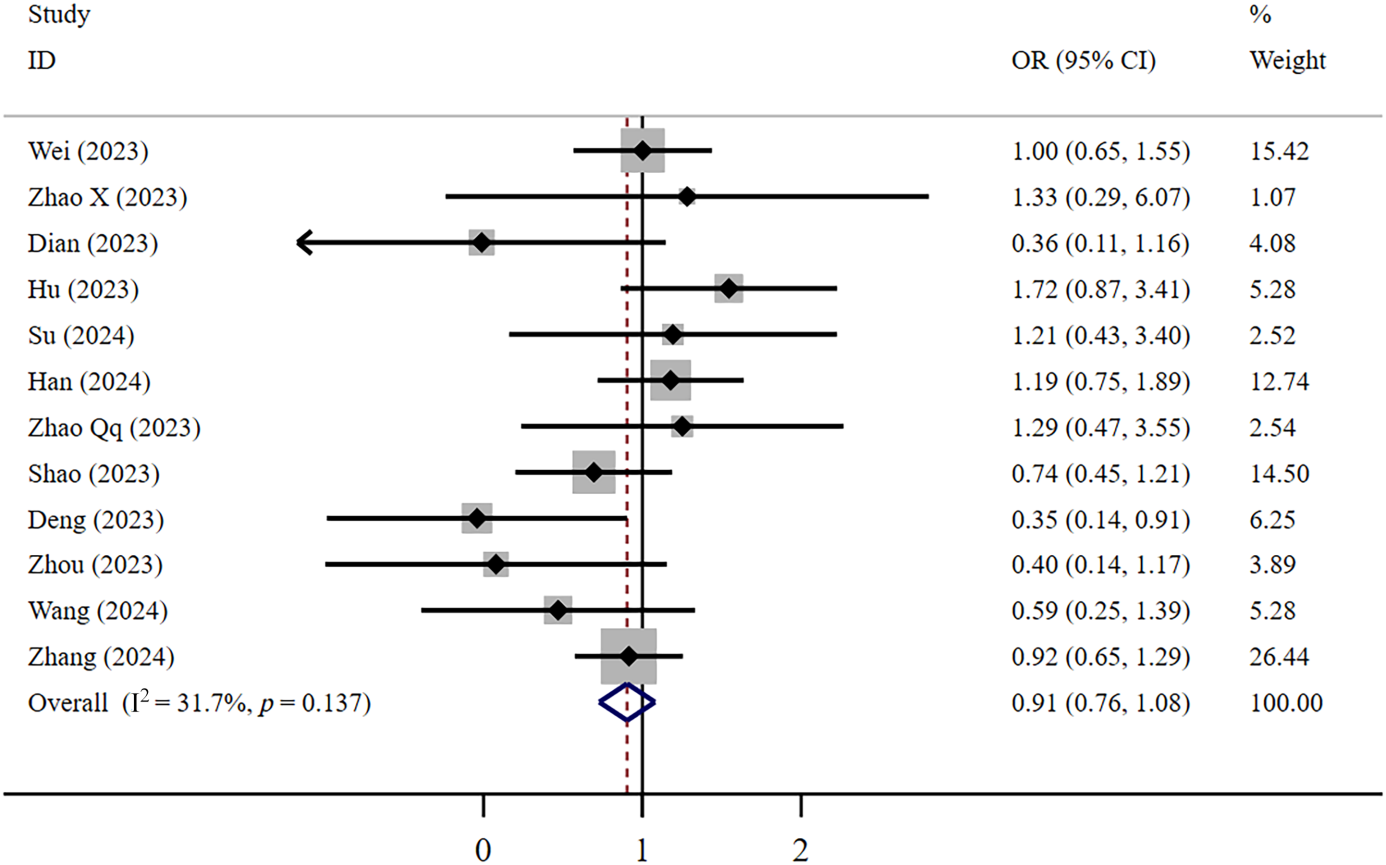
Mortality: FNC/Paxlovid.

**FIGURE 3:**
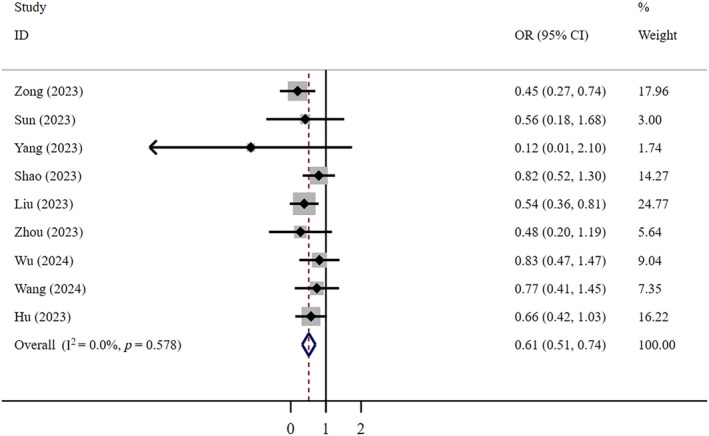
Mortality: FNC/control.

Low‐certainty evidence indicates that the therapeutic effect of FNC in reducing mortality rates is comparable to that of Paxlovid (OR = 0.91, 95% CI: 0.76–1.08; *I* ^2^ = 31.7%, *n* = 12) (Figure 2). Egger's test revealed no publication bias (*p* = 0.145). Low‐certainty evidence shows that compared to patients who did not receive antiviral treatment, patients who received FNC had a 39% lower mortality rate (OR = 0.61, 95% CI: 0.51–0.74; *I* ^2^ = 0; *n* = 9) (Figure 3). Egger's test indicated no publication bias (*p* = 0.777). The funnel plots of both aforementioned studies above show no significant asymmetry.

### 3.4 LOHS

**FIGURE 4:**
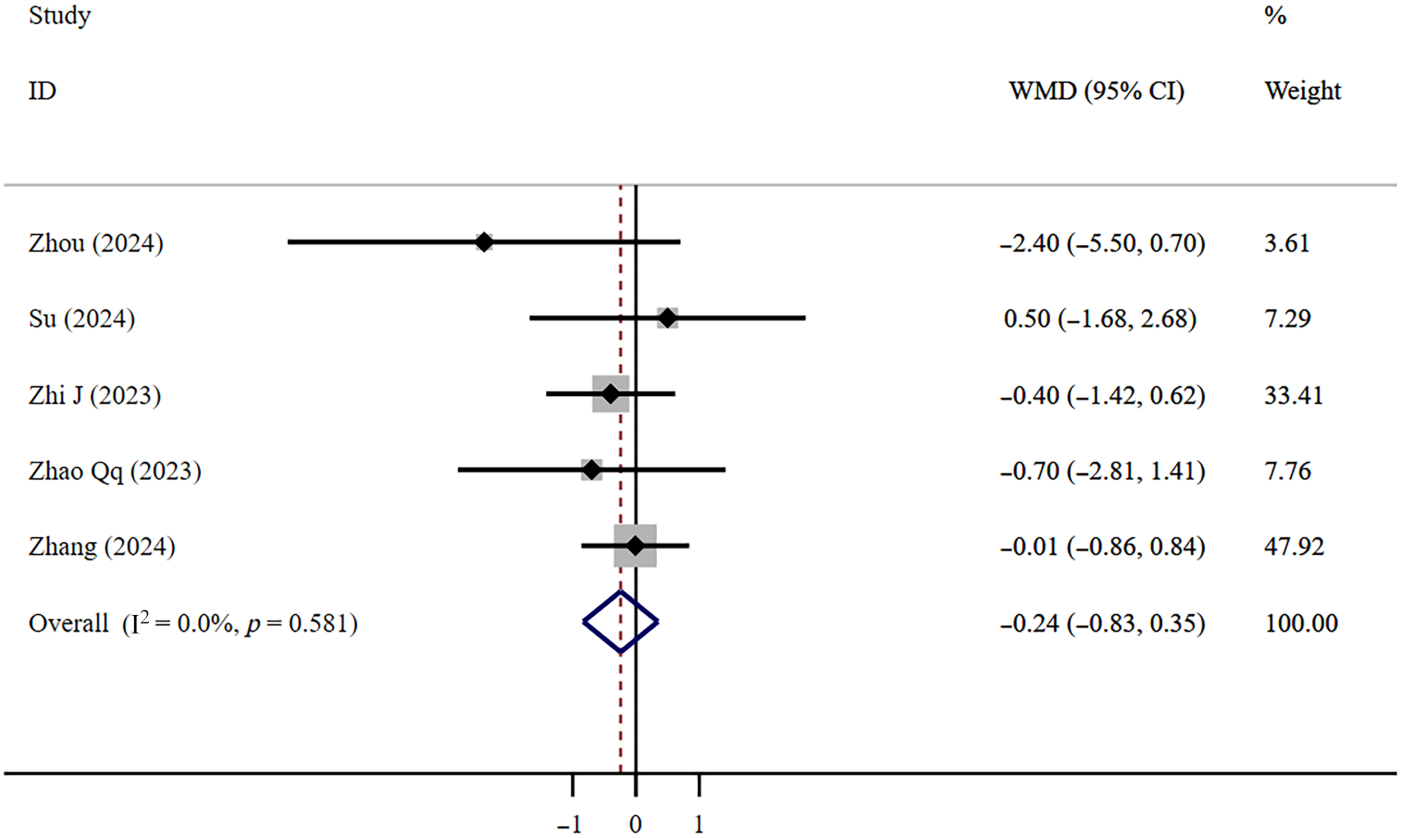
LOHS: FNC/Paxlovid.

**FIGURE 5:**
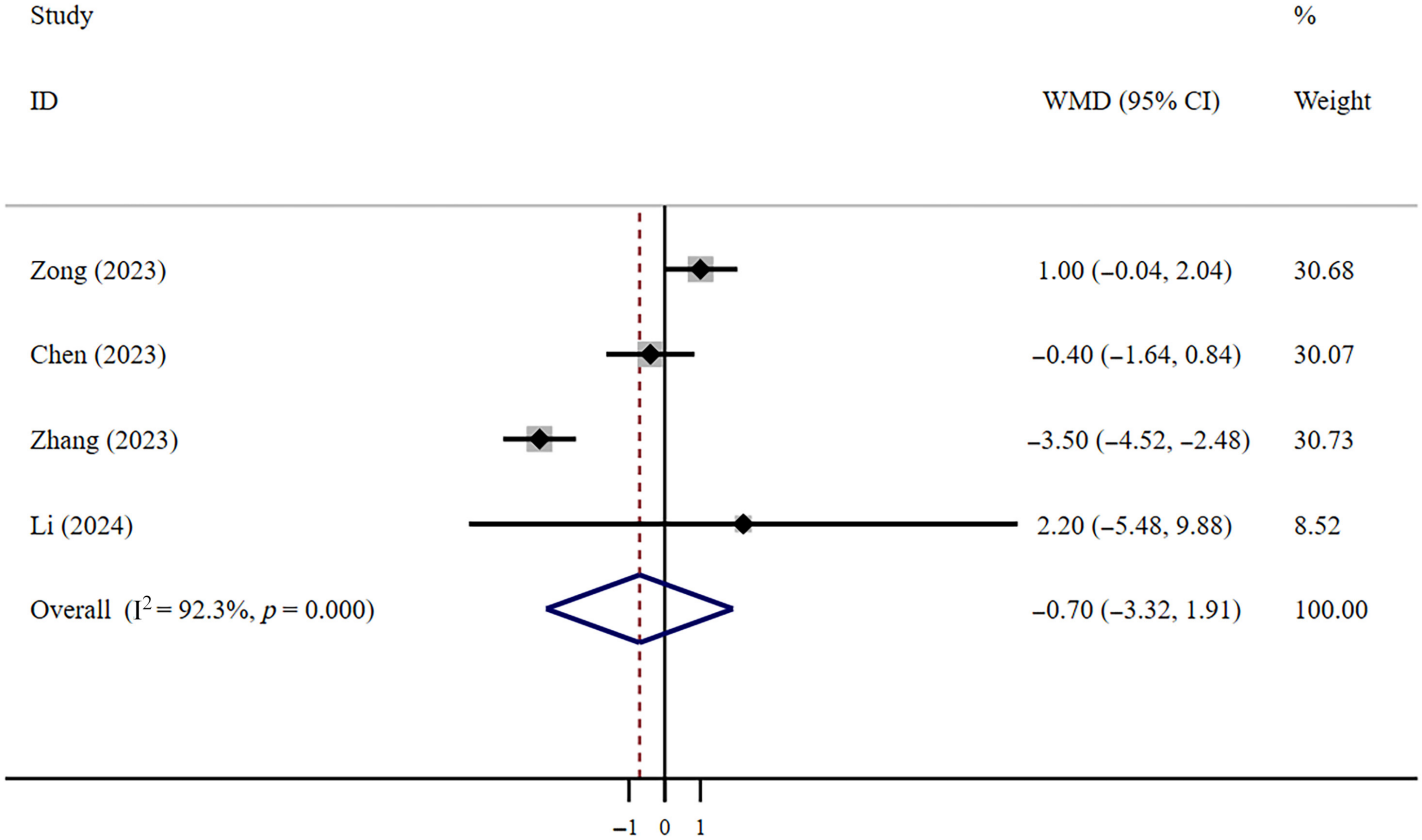
LOHS: FNC/control. Weights are from random effects analysis.

Low‐certainty evidence indicates that there is no significant difference in LOHS between FNC patients and Paxlovid patients (WMD = −0.24, 95% CI: −0.83 to 0.35; *I* ^2^ = 0, *n* = 5) (Figure 4). Egger's test indicated no publication bias (*p* = 0.918). Very low‐certainty evidence indicates that compared to patients who did not receive antiviral treatment, FNC did not effectively shorten the LOHS (WMD = −0.70, 95% CI: −3.32 to 1.91; *I* ^2^ = 92.3%, *n* = 4) (Figure 5). The Egger test revealed the absence of publication bias (*p* = 0.822).

### 3.5 T‐FNANC

**FIGURE 6:**
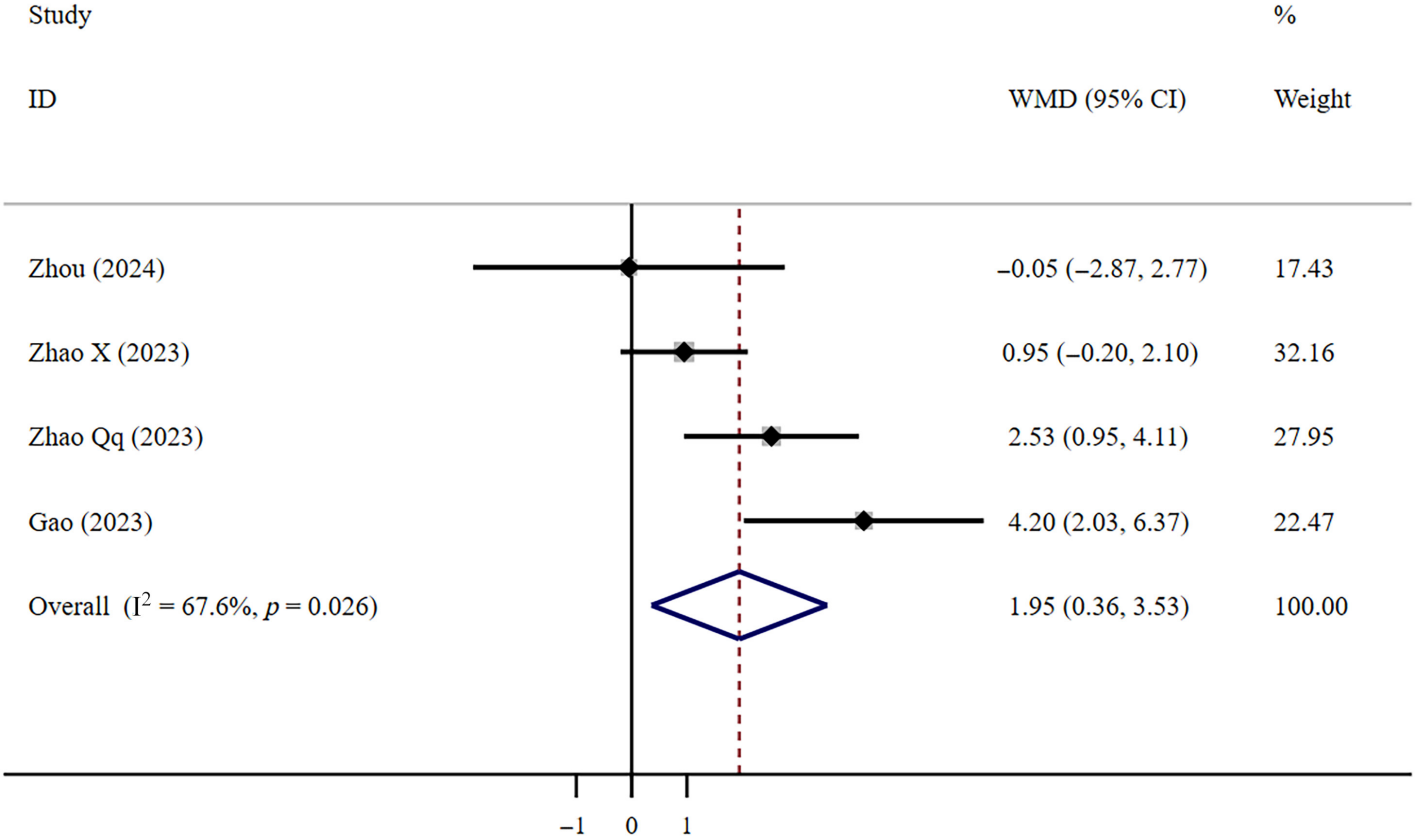
T‐FNANC: FNC/Paxlovid. Weights are from random effects analysis.

Very low‐certainty evidence suggests that compared to Paxlovid, FNC resulted in a longer T‐FNANC (WMD = 1.95, 95% CI: 0.36–3.53; *I* ^2^ = 67.6%, *n* = 4) (Figure 6). The Egger test revealed the absence of publication bias (*p* = 0.591).

**FIGURE 7:**
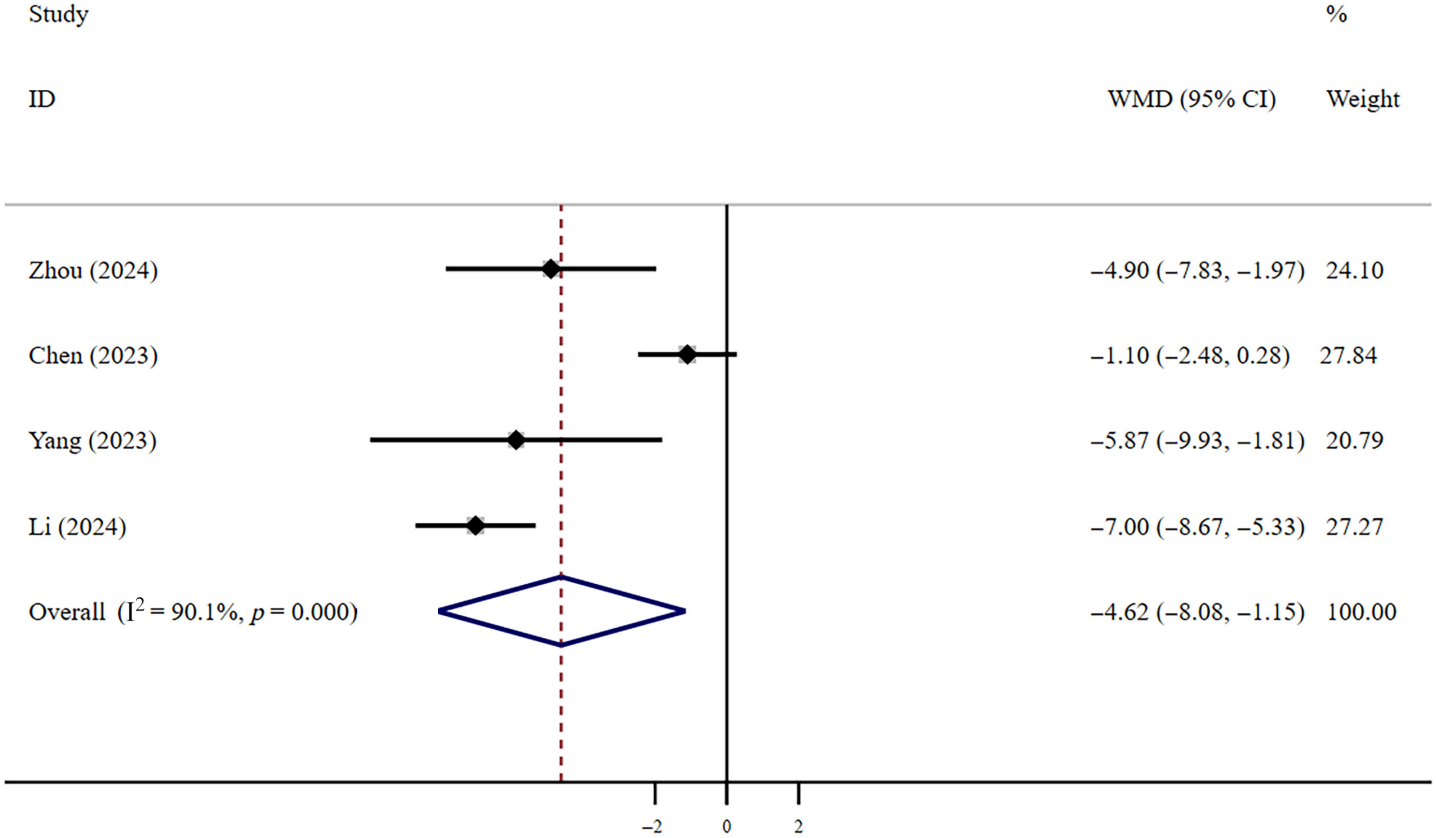
T‐FNANC: FNC/control. Weights are from random effects analysis.

Very low‐certainty evidence shows that compared to patients who did not receive antiviral treatment, patients who received FNC treatment had a shorter T‐FNANC (WMD = −4.62, 95% CI: −8.08 to −1.15; *I* ^2^ = 90.1%, *n* = 4) (Figure 7). The Egger test revealed the absence of publication bias (*p* = 0.078).

### 3.6 Composite Outcome

**FIGURE 8:**
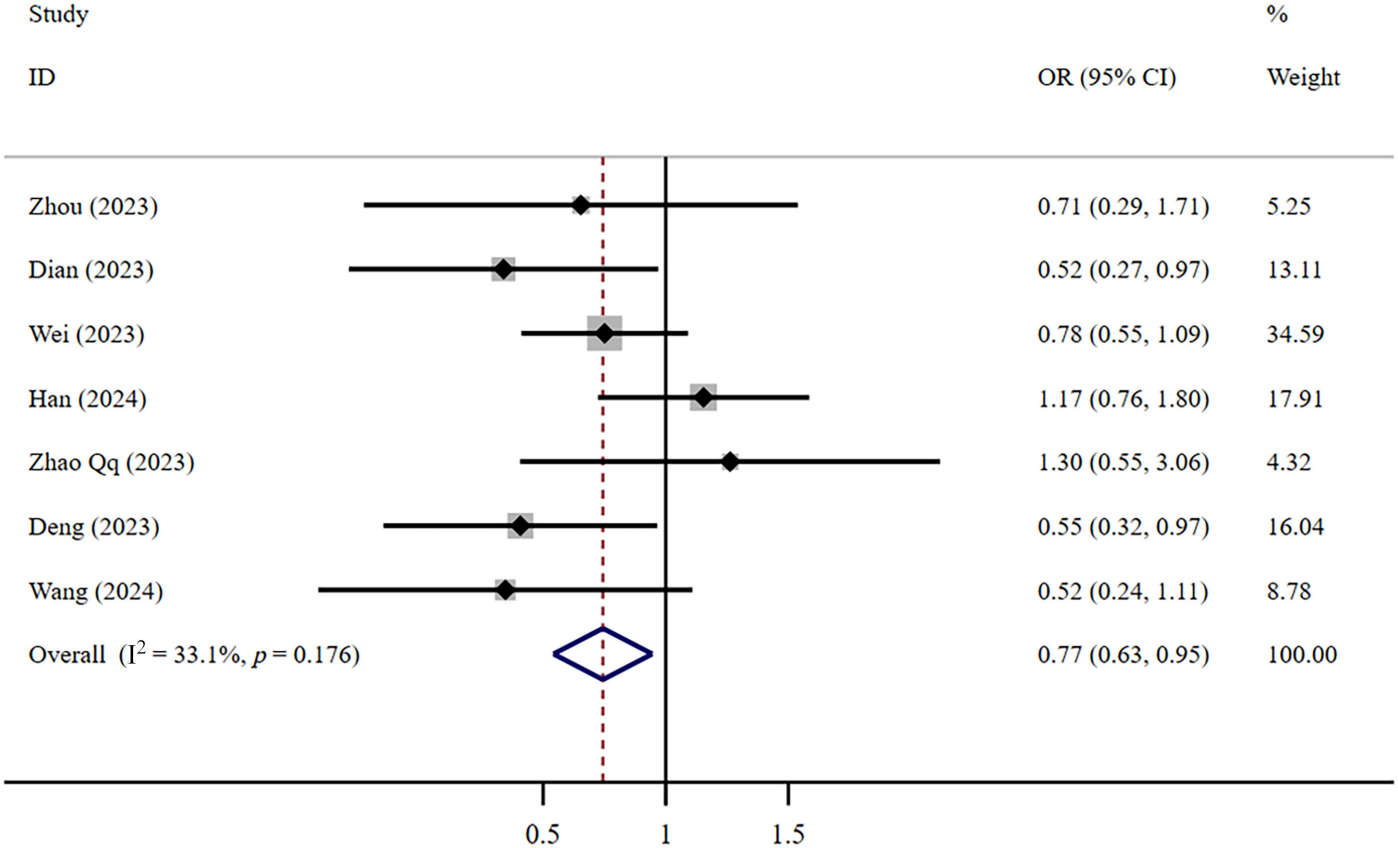
Composite outcome: FNC/Paxlovid.

**FIGURE 9:**
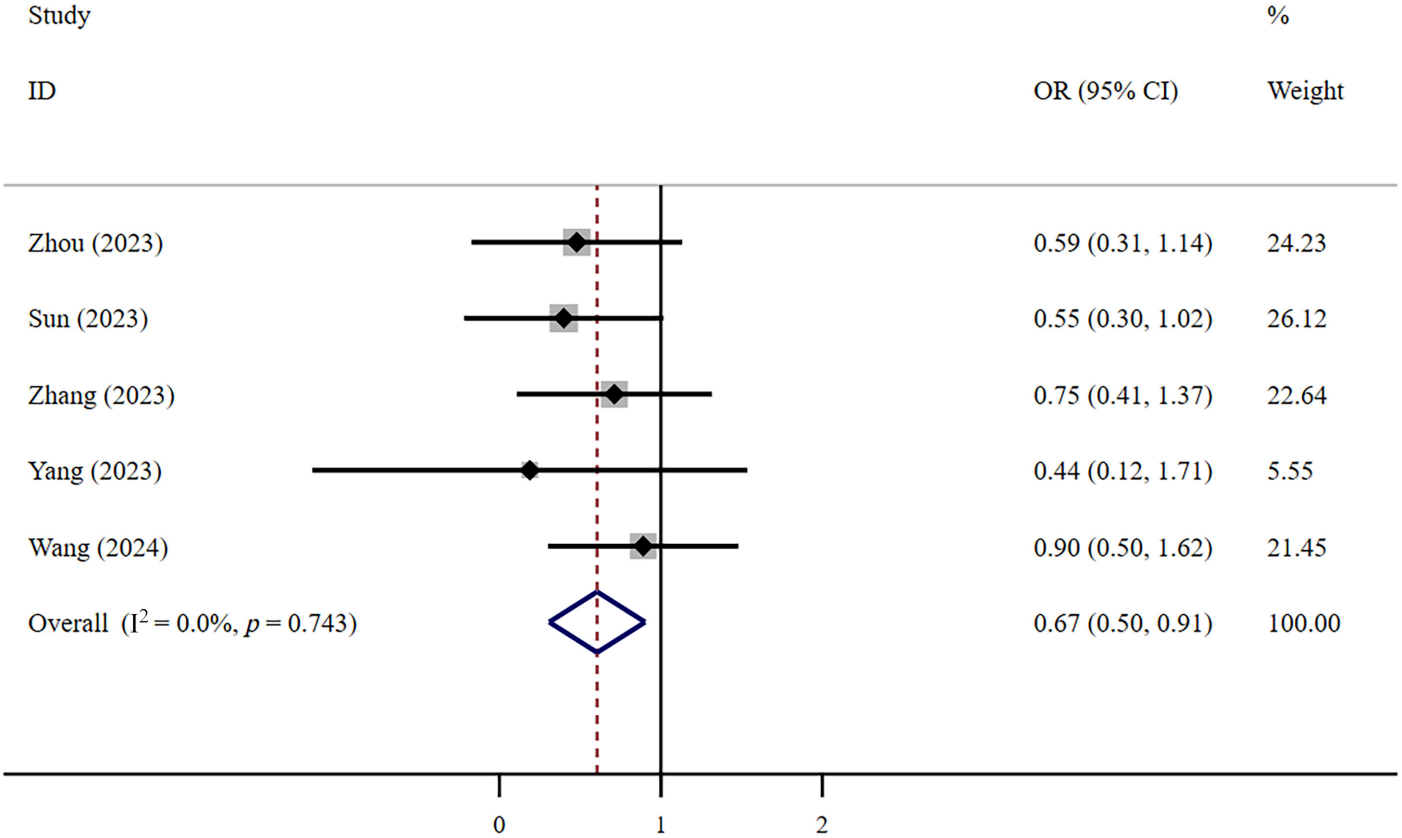
Composite outcome: FNC/control.

In terms of the composite outcome, very low‐certainty evidence suggests that FNC is less effective than Paxlovid (OR = 0.77, 95% CI: 0.63–0.95; *I* ^2^ = 33.1%, *n* = 7) (Figure 8). The Egger test revealed the absence of publication bias (*p* = 0.307). Low‐certainty evidence suggests that the therapeutic efficacy of FNC is also significantly less than that of supportive care (OR = 0.67, 95% CI: 0.50–0.91; *I* ^2^ = 0, *n* = 5) (Figure 9). The Egger test revealed the absence of publication bias (*p* = 0.923).

### 3.7 Adverse Events

**FIGURE 10:**
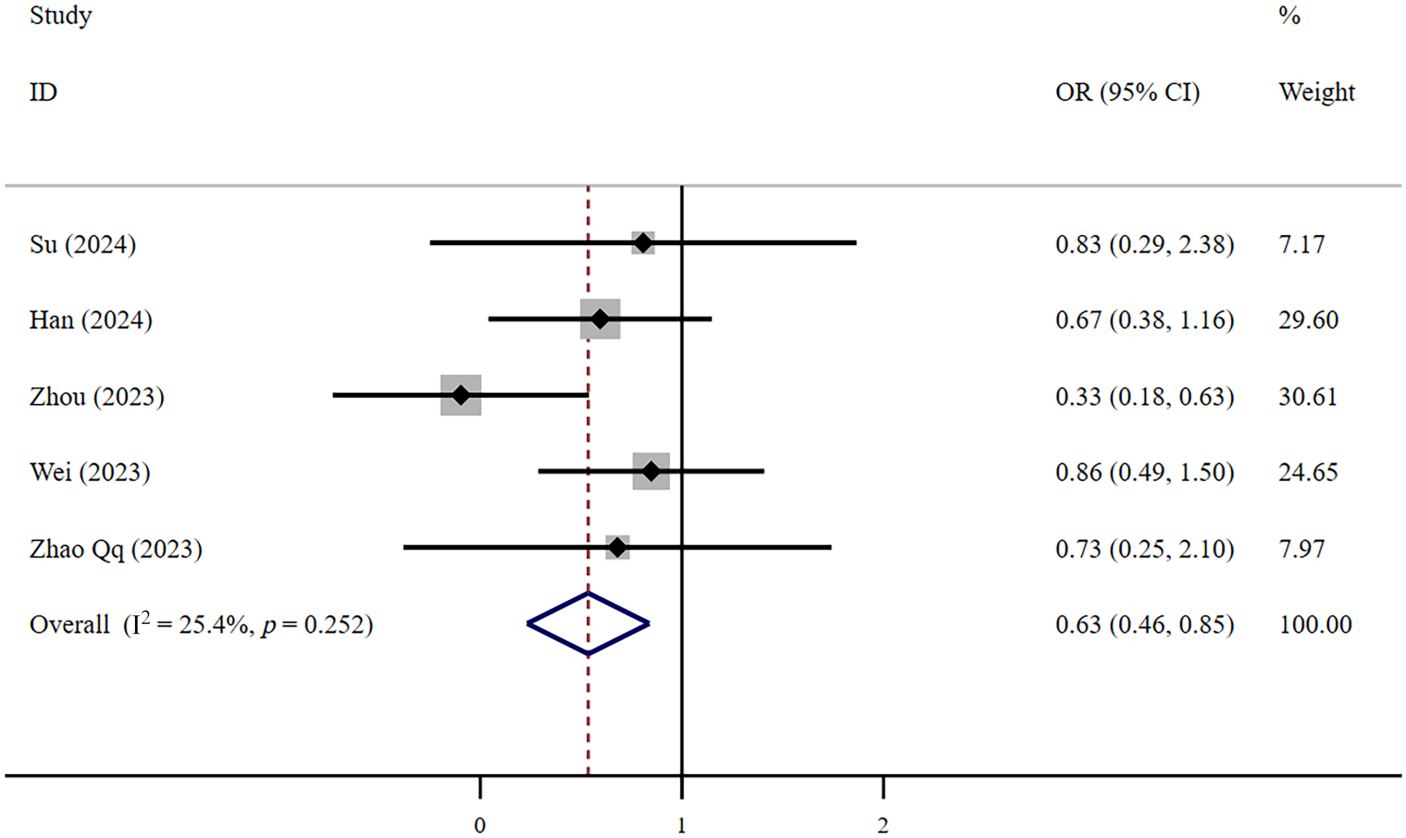
Adverse events: FNC/Paxlovid.

**FIGURE 11:**
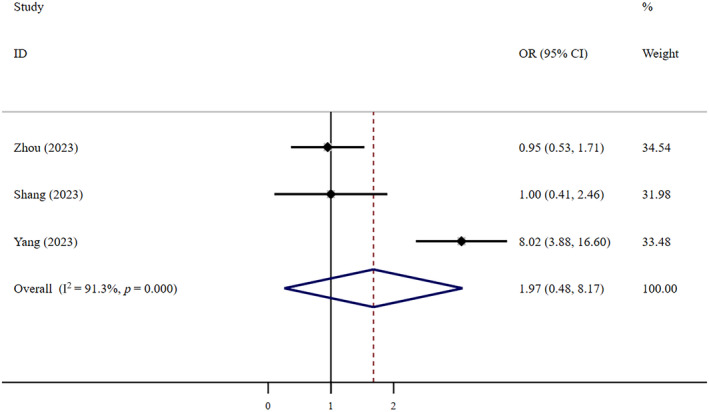
Adverse events: FNC/control. Weights are from random effects analysis.

Very low‐certainty evidence suggests that FNC was safer than Paxlovid (OR = 0.63, 95% CI: 0.46–0.85; *I* ^2^ = 25.4%, *n* = 5) (Figure 10). Low‐certainty shows that there was no statistically significant difference in safety between FNC and supportive therapy (OR = 1.97, 95% CI: 0.48–8.17; *I* ^2^ = 91.3%, *n* = 3) (Figure 11). Egger's test showed that neither of the two studies above had publication bias, with *p* values of 0.265 and 0.923, respectively.

## 4 Discussion

### 4.1 Comparison With Other Studies

This study represents the largest meta‐analysis to date of FNC therapy for COVID‐19, and it is also the first time that a head‐to‐head comparison of the efficacy of FNC and Paxlovid has been conducted using meta‐analysis methods. Wang et al. [37] systematically reviewed the efficacy of FNC and Paxlovid, concluding that FNC shorten T‐FNANC but may be less effective than Paxlovid. This finding aligns with our conclusion: We found that compared to supportive therapy, FNC indeed shorten T‐FNANC, but patients receiving FNC had a longer T‐FNANC compared to Paxlovid. Regarding mortality, Wang et al. [37] found that FNC reduce mortality compared to supportive therapy, a result consistent with our analysis. However, their study did not compare the difference in mortality between FNC and Paxlovid, while we found no significant difference in mortality between the two drugs. We analyzed the results of composite outcome and found that the use of FNC reduces the occurrence of composite outcome compared to supportive therapy. In terms of safety outcomes, the safety of FNC has been confirmed in the treatment of acquired immune deficiency syndrome [3]. Wang et al.'s [37] study also found no statistically significant difference in safety between FNC and supportive therapy (OR 1.26, 95% CI: 0.59–2.70), which is similar to our findings, Chen et al.'s [38] study confirmed this conclusion. Similar to the aforementioned studies [37, 38], we found that adverse events with FNC were significantly fewer than with Paxlovid, indicating better safety. We evaluated the certainty of all evidence using the GRADE evidence system and found that the overall certainty of evidence is low, with most being of low certainty and a few being of very low certainty, which leads us to adopt a cautious attitude towards the study results. These differences between our study and others contribute to a more comprehensive understanding of FNC's protective role in real‐world scenarios, allowing for a more thorough evaluation of FNC's protective effects.

### 4.2 Heterogeneity of Included Studies

The quality differences among the included studies were minimal, with only one study rated as serious, while the others were of moderate quality. These outcomes all demonstrated statistically significant protective effects of FNC on various outcomes, except for the outcome of T‐FNANC. Exclusion of studies deemed to have a serious risk of bias did not significantly alter the results for each outcome.

We did not find any signs of publication bias in most outcomes. However, in the comparison between FNC and supportive therapy, there was substantial heterogeneity in the outcomes of LOHS and T‐FNANC, suggesting potential publication bias. Therefore, the summary estimates of these two outcomes should be interpreted critically.

### 4.3 Limitation

The studies included in our analysis were all conducted in China, which suggests that the findings may not necessarily be generalizable to other countries. Given the GRADE ratings were poor, it is important to interpret these findings with caution.

## 5 Conclusion

Although our study suggest that FNC may offer considerable benefits and has relatively good safety, the low certainty of the evidence makes us cautious about this conclusion. Further research with larger cohorts and higher quality is needed to confirm these preliminary findings.

## Author Contributions

Study design: Wentao Zhang, Tao Dong, and Yuna Liu. Data collection: Wentao Zhang, Tingting Wu, Tao Dong, Yongxiang Ge, Qi Yang, and Jia Xu. Data analysis: Wentao Zhang and Tao Dong. Writing: Wentao Zhang, Tingting Wu, and Yuna Liu. All authors have reviewed the manuscript.

## Ethics Statement

The authors have nothing to report.

## Conflicts of Interest

The authors declare no conflicts of interest.

## Supporting information


**Data S1** Supporting Information

## Data Availability

The data that support the findings of this study are available from the corresponding author (Yuna Liu) upon reasonable request.
