# Supplementary material for: Efficacy and Safety of Azvudine in Patients With COVID‐19 in China: A Meta‐Analysis of Observational Studies
Source: Clin Respir J. 2024 Jul 12;18(7):e13798. doi: 10.1111/crj.13798 (PMC11240111; doi:10.1111/crj.13798)
Supplement: Supplementary file 1 — Data S1 Supporting Information [file CRJ-18-e13798-s001.docx]

Supplementary Material

Table 1 GRADE analyses-FNC/ Paxlovid

| **Outcome** | **Study(N)** | **Quality assessment** | | | | | **Quality** | **Importance** |
| --- | --- | --- | --- | --- | --- | --- | --- | --- |
|  |  |  | **Inconsistency** | **Indirectness** | **Imprecision** | **Other considerations** |  |  |
| Mortality | 12 | no serious | no serious | no serious | no serious | none | Low | Critical |
| Composite outcome | 7 | no serious | no serious | no serious | no serious | none | Low | Important |
| LOHS | 5 | no serious | no serious | no serious | no serious | none | Low | Important |
| T-FNANC | 4 | no serious | no serious | no serious | serious^*^ | none | Very low | Important |
| Adverse events | 5 | no serious | no serious | no serious | serious^*^ | none | Very low | Important |

^* Downgraded by one level due to imprecision. This is based on the consideration that these results have a relatively wide confidence interval, which introduces a significant degree of uncertainty to the results.^

Table 2 GRADE analyses-FNC/Control

| **Outcome** | **Study(N)** | **Quality assessment** | | | | | **Quality** | **Importance** |
| --- | --- | --- | --- | --- | --- | --- | --- | --- |
|  |  |  | **Inconsistency** | **Indirectness** | **Imprecision** | **Other considerations** |  |  |
| Mortality | 9 | no serious | no serious | no serious | no serious | none | Low | Critical |
| Composite outcome | 5 | no serious | no serious | no serious | no serious | none | Low | Important |
| LOHS | 4 | no serious | no serious | no serious | Serious^*^ | none | Very low | Important |
| T-FNANC | 4 | no serious | no serious | no serious | Serious^*^ | none | Very low | Important |
| Adverse events | 3 | no serious | no serious y | no serious | no serious | none | Low | Important |

^*^ ^Downgraded by one level due to imprecision. This is based on the consideration that these results have a relatively wide confidence interval, which introduces a significant degree of uncertainty to the results.^
